# Applying WHO2013 diagnostic criteria for gestational diabetes mellitus reveals currently untreated women at increased risk

**DOI:** 10.1007/s00592-023-02148-2

**Published:** 2023-07-18

**Authors:** Cathrine Munk Scheuer, Dorte Møller Jensen, H. David McIntyre, Lene Ringholm, Elisabeth Reinhardt Mathiesen, Celina Pforr Korsgård Nielsen, Rúna Louise Mortansdóttir Nolsöe, Julie Milbak, Thore Hillig, Peter Damm, Martin Overgaard, Tine Dalsgaard Clausen

**Affiliations:** 1 Department of Gynaecology and Obstetrics, Nordsjællands Hospital Hillerød, Hillerød, Denmark; 2 Steno Diabetes Center Odense, Odense University Hospital, Odense, Denmark; 3 Department of Gynecology and Obstetrics, Odense University Hospital, Odense, Denmark; 4 Department of Clinical Research, University of Southern Denmark, Odense, Denmark; 5 Mater Research, Faculty of Medicine, The University of Queensland, Brisbane, Australia; 6 Department of Endocrinology and Metabolism, Center for Pregnant Women with Diabetes, Rigshospitalet, Copenhagen, Denmark; 7 Department of Clinical Medicine, University of Copenhagen, Copenhagen, Denmark; 8 Department of Endocrinology and Nephrology, Nordsjællands Hospital Hillerød, Hillerød, Denmark; 9 Department of Clinical Biochemistry, Nordsjællands Hospital Hillerød, Hillerød, Denmark; 10 Center for Pregnant Women with Diabetes, Department of Obstetrics, Rigshospitalet, Copenhagen, Denmark; 11 Department of Clinical Biochemistry, Odense University Hospital, Odense, Denmark

**Keywords:** Diagnosis, Gestational diabetes mellitus, IADPSG, Outcomes, Prevalence, WHO

## Abstract

**Aims:**

To estimate the prevalence of gestational diabetes mellitus (GDM) in a Danish cohort comparing the current Danish versus the WHO2013 diagnostic criteria, and to evaluate adverse pregnancy outcomes among currently untreated women in the gap between the diagnostic thresholds.

**Methods:**

Diagnostic testing was performed by a 75 g oral glucose tolerance test (OGTT) at 24–28 weeks’ gestation in a cohort of pregnant women. GDM diagnosis was based on the current Danish criterion (2-h glucose ≥ 9.0 mmol/L, GDM_DK_) and on the WHO2013 criteria (fasting ≥ 5.1, 1 h ≥ 10.0 or 2 h glucose ≥ 8.5 mmol/L, GDM_WHO2013_). Currently untreated women fulfilling the WHO2013 but not the Danish diagnostic criteria were defined as New-GDM-women (GDM_WHO2013_-positive and GDM_DK_-negative). Adverse outcomes risks were calculated using logistic regression.

**Results:**

OGTT was completed by 465 women at a median of 25.7 weeks’ gestation. GDM_DK_ prevalence was 2.2% (*N* = 10) and GDM_WHO2013_ 21.5% (*N* = 100). New-GDM was present in 19.4% (*N* = 90), of whom 90.0% had elevated fasting glucose. Pregnancies complicated by New-GDM had higher frequencies of pregnancy-induced hypertension (13.3% vs 4.1%, *p* = 0.002), large-for-gestational-age infants (22.2% vs 9.9%, *p* = 0.004), neonatal hypoglycaemia (8.9% vs 1.9%, *p* = 0.004) and neonatal intensive care unit admission (16.7% vs 5.8%, *p* = 0.002) compared to pregnancies without GDM.

**Conclusions:**

GDM prevalence increased tenfold when applying WHO2013 criteria in a Danish population, mainly driven by higher fasting glucose levels. Untreated GDM in the gap between the current Danish and the WHO2013 diagnostic criteria resulted in higher risks of adverse pregnancy outcomes.

**Supplementary Information:**

The online version contains supplementary material available at 10.1007/s00592-023-02148-2.

## Introduction

The landmark study Hyperglycemia and Adverse Pregnancy Outcome (HAPO) showed a positive, linear association between maternal glucose and adverse pregnancy outcomes [1]. This led to a consensus recommendation for universal screening and uniform international diagnostic criteria for gestational diabetes mellitus (GDM), which was endorsed by the WHO in 2013 (WHO2013 diagnostic criteria). The recommendation included thresholds of fasting plasma glucose (FPG) ≥ 5.1, 1 h ≥ 10.0 and/or 2 h ≥ 8.5 mmol/L using a 75 g oral glucose tolerance test (OGTT) [2, 3]. Shifting to these new diagnostic criteria, which were lower than those previously used in many countries, decreased the biochemical thresholds for GDM diagnosis and thus resulted in substantial increases in GDM prevalence [4–6]. Despite the desire for internationally consistent diagnostic criteria, questions have been raised as to whether the principle of “one size fits all” should apply to diagnosing GDM regardless of ethnic and genetic differences – a challenge in relation to all measured glucose values but in some populations, particularly the FPG threshold [7]. This debate motivated further international suggestions on modified fasting thresholds, including a threshold of 5.6 mmol/L as a clinically relevant cut-off when predicting large-for-gestational-age (LGA) infants in pregnant women from the Danish Odense Child Cohort (OCC) [8].

Recently, the Gestational Diabetes Mellitus Trial of Diagnostic Detection Thresholds Study (GEMS) explored effects of treatment using the lower WHO2013 diagnostic criteria compared to higher diagnostic thresholds (FPG ≥ 5.5 or 2 h ≥ 9.0 mmol/L) [9]. Although at the level of the whole population, the primary outcome, LGA, was not significantly improved by treatment of women diagnosed by these lower thresholds, secondary analyses showed that women who had glucose values in the gap between the lower and higher thresholds benefited significantly from treatment.

In Denmark, GDM screening is risk-factor-based, followed by a 2 h 75 g OGTT with a 2 h glucose ≥ 9.0 mmol/L as the sole diagnostic criterion [10]. In contrast to universal screening, which offers all women diagnostic testing, risk-factor-based screening only includes high-risk women. This screening strategy and diagnostic criterion are based on a study from 2003, but the risk-factor profile of Danish pregnant women may have changed since, raising questions about the current efficacy of this strategy [11]. No prior studies have evaluated the WHO2013 criteria in a prospective Danish cohort. Our primary aim was to estimate the GDM prevalence in a Danish cohort, comparing the current Danish diagnostic criterion versus the WHO2013 criteria, and secondly, to evaluate adverse pregnancy outcomes in women with glucose values in the gap between the two sets of diagnostic criteria, who are currently left untreated.

## Methods

### Study design and population

In this single centre observational cohort study, we introduced universal screening for GDM at 24–28 weeks’ gestation. The study was approved by the local Committee on Health Research Ethics (H-19001203) and Data Protection Agency (P-2019-89) and was conducted in accordance with the Helsinki Declaration. Written informed consent was obtained from all participants.

All women attending the routine first-trimester ultrasonography scan at Nordsjællands Hospital Hillerød from October 2019 to March 2020 were assessed for enrolment. Women were eligible if they had at least one living fetus at gestational age 11 + 2 – 13 + 6 (weeks + days). Exclusion criteria were: age < 18 years, pre-existing type 1 or 2 diabetes, psychiatric conditions severe enough to hinder informed consent or completion of OGTT, previous bariatric surgery, no social security number or insufficient Danish or English language skills. Women who declined participation with OGTT were invited to participate with medical record data only.

### Present GDM screening and diagnostic testing in Denmark

The present Danish screening program for GDM is risk-factor based, including: GDM in a previous pregnancy, prior birth of a macrosomic infant (≥ 4,500 g), pre-pregnancy body mass index (BMI) ≥ 27 kg/m^2^, family history of diabetes, polycystic ovarian syndrome or a current multiple pregnancy [10]. Women presenting at least one of these risk factors are offered a diagnostic test for GDM at 24–28 weeks’ gestation. Women with GDM in a previous pregnancy or at least two of the other risk factors are additionally offered early testing (10–20 weeks’ gestation). Furthermore, glycosuria detected at any time during pregnancy and, based on individual judgement, other clinical findings (e.g. ultrasonic estimated fetal weight >  + 22% of the mean or polyhydramnios) are indicative for diagnostic testing.

In the present study, all women were offered GDM testing at 24–28 weeks’ gestation regardless of risk-factor profile. The diagnostic test consisted of a 2 h 75 g OGTT with assessment of venous plasma glucose at fasting, 1 and 2 h, fasting samples being drawn between 8.00 and 9.00 AM. Venous blood was sampled in sodium-fluoride-EDTA-citrate (FC-Mix) tubes and stored at room temperature a maximum of four hours before centrifugation for 10 min at 2500 g. Centrifuged tubes were stored at 2–8 °C until analysis, which was performed within 4 h from centrifugation using Dimension Vista® 1500, Siemens Medical Solutions Diagnostics.

### Study groups and pregnancy care

The study population was divided into three groups based on OGTT glucose values fulfilling:

1. The current Danish diagnostic criterion for GDM (GDM_DK_-women): 2 h glucose ≥ 9.0 mmol/L
2. The WHO2013 criteria but not the Danish (New-GDM-women): fasting glucose ≥ 5.1, 1 h glucose ≥ 10.0 and/or 2 h glucose ≥ 8.5–8.9 mmol/L
3. Neither the WHO2013 diagnostic nor the Danish criteria for GDM (No-GDM-women): fasting glucose < 5.1, 1 h glucose < 10.0 and 2 h glucose < 8.5 mmol/L

Only women fulfilling the current Danish diagnostic criterion received treatment for GDM, while New-GDM-women remained untreated besides standard pregnancy care. Participants and clinicians were blinded to the fasting and 1 h glucose values but not the 2 h.

### Data collection and covariates

Baseline characteristics were assessed by a personal interview at inclusion and all data on maternal and neonatal outcomes were collected from medical records. BMI was calculated from self-reported pre-pregnancy weight and height measured at inclusion. Family history of diabetes was defined as presence of diabetes in 1st or 2nd degree relatives. Glycosuria was registered if a dipstick urine test was ≥ 5.5 mmol/L (+ 1) on Siemens Clinitek Status Uristix. First generation country of origin was categorised as Denmark vs not Denmark, and relationship status as married/de-facto relationship vs no partner. Higher education included vocational education, short-, middle- and long-cycled higher educations, and employed comprised employment at the time of inclusion. Women smoking at the time of inclusion were reported as current smokers. Chronic hypertension was defined as blood pressure ≥ 140/90 mmHg predating pregnancy or recognised < 20 weeks’ gestation [12].

Pregnancy-induced hypertension included gestational hypertension, preeclampsia (including Haemolysis Elevated Liver enzymes and Low Platelet count syndrome) or eclampsia [12]. Postpartum haemorrhage was defined as estimated blood loss ≥ 1,000 mL within 24 h after delivery. Gestational age and sex-adjusted birth weight z-score was calculated (20) and LGA and small-for-gestational-age infants were defined as birth weight > 90th percentile and < 10th percentile, respectively. Occurrence of shoulder dystocia, neonatal nerve injury or bone fracture defined a composite complication outcome. Neonatal hypoglycaemia was defined as plasma glucose < 2.5 mmol/L at 2 h of life [13]. The remaining baseline and outcome variables were noted as described in the medical record.

### Statistical analyses

Data are given as the percentage (number) for categorical data and mean (standard deviation) or median (25–75 percentile) for continuous variables as appropriate. For categorical variables, comparisons of groups were assessed by Fisher’s exact test, and for continuous variables by Student’s T-test if normally distributed data or Mann–Whitney *U* test when skewed.

Original power calculations were based on a GDM prevalence of 3% among Danish women and an intention to detect ≥ 1.5% change in prevalence. With a power of 90% and a significance level of 5% the resulting sample size reached 1,647. Due to the COVID-19 pandemic, only 465 participants were included in the study.

Sensitivity analyses were performed comparing 1) baseline characteristics from medical record data of study participants vs non-participants and 2) pregnancy outcomes of untreated women (New-GDM-women vs No-GDM-women).

For secondary analyses comparing maternal and neonatal outcomes between groups, we corrected for multiple testing using the Benjamini-Hochberg (BH) equation i/m*Q setting *Q* to 5% [14, 15]. The resulting corrected significance levels (i.e. the BH critical values) were 0.017 for the comparisons of New-GDM and No-GDM women, and 0.014 for the sensitivity analysis of pregnancy outcomes among untreated women (Online Resource 1).

The four adverse maternal and neonatal outcomes that differed significantly between New-GDM and No-GDM-women were further evaluated in explorative logistic regression analyses. In adjusted analyses, pregnancy-induced hypertension was adjusted for maternal BMI, age and chronic hypertension; Large-for-gestational-age for maternal BMI, country of origin, educational level, smoking, parity and infant sex; neonatal hypoglycaemia for maternal BMI; and neonatal intensive care unit (NICU) admission for maternal BMII and age.”

Missing data were excluded. All analyses were performed using IBM SPSS Statistics, version 25.0.

## Results

**Fig. 1:**
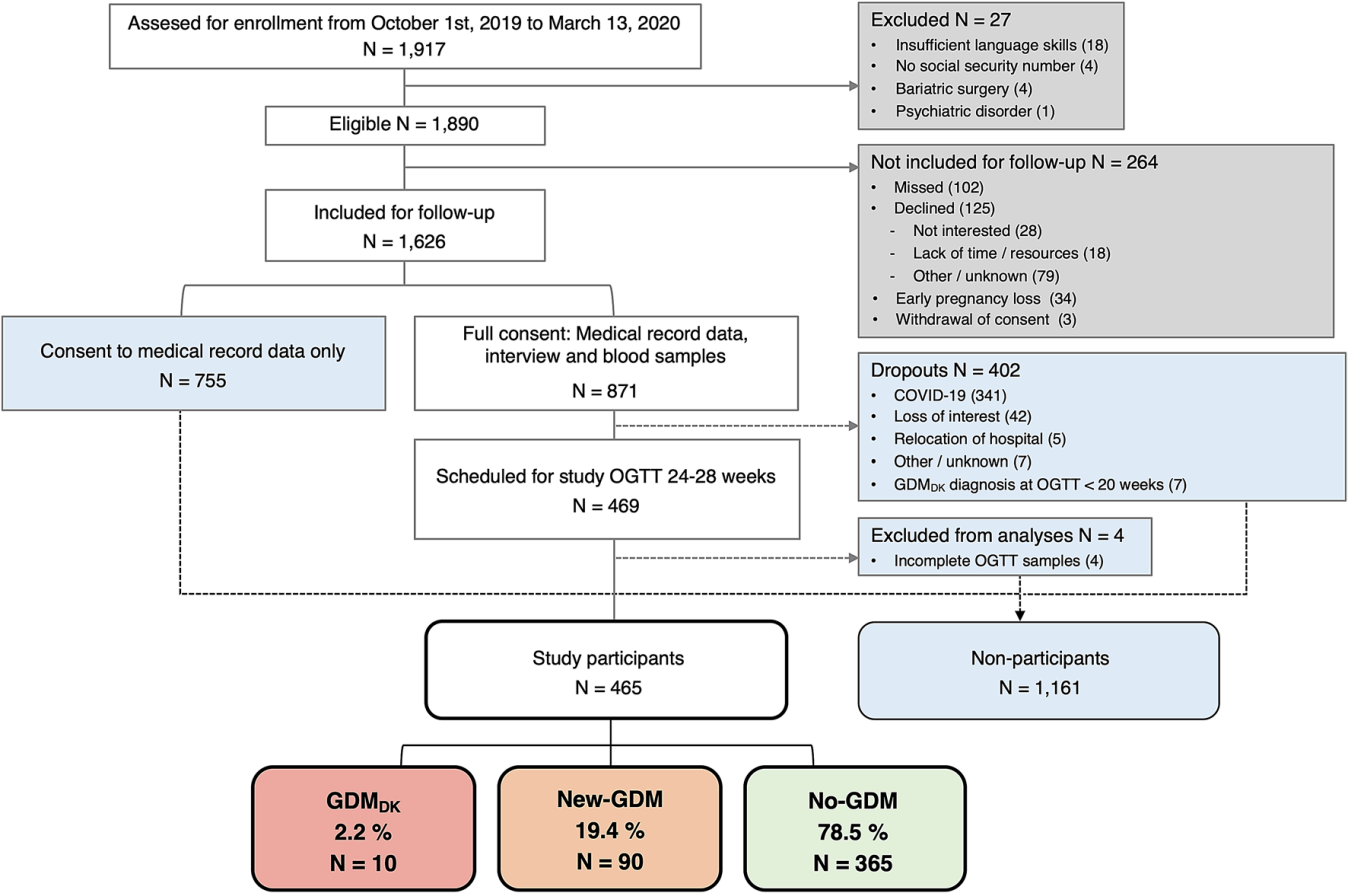
Flowchart of study design and participation rates. OGTT: oral glucose tolerance test

Of 1,890 women eligible for inclusion, 1,626 were included for follow-up of whom 871 gave full consent, while 755 gave consent to contribute only with medical record data (Fig. 1). Of the 871 women with full consent, 465 completed the study OGTT at 24–28 weeks’ gestation. These 465 women were defined as study participants, whereas the remaining women who were included without a study OGTT were defined as non-participants (*N* = 1,161).

The COVID-19 pandemic, which shut down all clinical research in Denmark in March 2020, was responsible for 84.8% (341/402) of missing OGTTs, and the duration of restrictions made it impossible to resume inclusion (Fig. 1). Among the 1,626 women included for follow-up, the main covariates with missing data were family history of diabetes (*N* = 111) and country of origin (*N* = 110). Otherwise, missing data were in general low (details summarised in the legend of Online Resource 2).

### Baseline characteristics

Characteristics of the study cohort are shown in Table 1. In total, 62.4% had at least one risk factor fulfilling the indications for risk-factor-based screening in Denmark. Eight of ten women with GDM_DK_ had risk factors, while two were diagnosed only as a result of universal screening. New-GDM-women had higher frequencies of risk factors for GDM compared to No-GDM-women.

**Table 1:** Baseline characteristics by groups based on OGTT at 24–28 weeks’ gestation

	All	GDM_DK_	New-GDM	No-GDM
Participants% (N)	100.0% (465)	2.2% (10)	19.4% (90)	78.5% (365)
Age at delivery (years)	31.9 (4.5)	34.3 (3.8)	31.4 (4.6)	32.0 (4.4)
*Risk factors for GDM*				
GDM in previous pregnancy	0.9% (4)	20.0% (2)	2.2% (2)	0% (0)
Body mass index ≥ 27 kg/m^2^	26.0% (121)	60.0% (6)	45.6% (41)	20.3% (74)
Previous birth of child ≥ 4,500 g	2.2% (10)	0.0% (0)	2.2% (2)	2.2% (8)
Family history of diabetes	38.0% (176)	33.3% (3)	42.7% (38)	37.0% (135)
Polycystic ovarian syndrome	4.1% (19)	20.0% (2)	3.3% (3)	3.8% (14)
Multiple pregnancy	0.9% (4)	10.0% (1)	0% (0)	0.8% (3)
Glycosuria	12.7% (59)	30.0% (3)	18.9% (17)	10.7% (39)
≥ 1 risk factor for GDM	62.4% (290)	80.0% (8)	77.8% (70)	58.1% (212)
≥ 2 risk factors for GDM	19.4% (90)	70.0% (7)	31.1% (28)	15.1% (55)
Pre-gestational body mass index (kg/m^2^) ^a^	23.9 (21.5 – 27.2)	30.1 (24.1 – 33.0)	26.2 (23.2 – 30.1)	23.3 (21.3 – 26.2)
Parous (≥ 1 prior births)	61.3% (285)	80.0% (8)	57.8% (52)	61.6% (225)
Country of origin: Denmark	81.0% (376)	90.0% (9)	81.1% (73)	80.8% (294)
Higher education	87.4% (403)	90.0% (9)	82.0% (73)	88.7% (321)
Employed	77.8% (360)	80.0% (8)	72.2% (65)	79.1% (287)
Married / de-facto relationship	99.1% (461)	100.0% (10)	98.9% (89)	99.2% (362)
Current smoker	3.9% (18)	0% (0)	8.9% (8)	2.7% (10)
Chronic hypertension	0.6% (3)	0% (0)	1.1% (1)	0.5% (2)

*OGTT* Oral glucose tolerance test, *GDM* Gestational diabetes mellitus, *GDM*_*DK*_ Women diagnosed with GDM by the Danish criterion (2 h glucose ≥ 9.0 mmol/L), *New-GDM* Women GDM_WHO2013_ positive and GDM_DK_ negative, *No-GDM* GDM_WHO2013_ and GDM_DK_ negative

Data are given as mean (SD) or percentages (N) unless otherwise stated

^a^Data are median (25–75 percentiles), as data were not normally distributed

Study participants more often had risk factors for GDM compared to non-participants (63.7% vs 55.4%), with family history of diabetes and glycosuria being significantly more frequent (Online Resource 2). Study participants also had higher BMI, more OGTTs performed on clinical indication and a higher frequency of GDM_DK_ diagnosis before 20 weeks’ gestation.

### GDM prevalence

Of 465 women completing the study OGTT, 10 (2.2%) had GDM_DK_ (Table 2). A total of 100 women (21.5%) had GDM_WHO2013_, of whom 90 (19.4%) were classified as New-GDM. The remaining 365 women (78.5%) met neither the Danish nor the WHO2013 criteria and were classified as No-GDM-women. Among the New-GDM-women, 90.0% (81/90) met the WHO2013 FPG threshold. Hereof 74.4% (67/90) had GDM based solely on the FPG value. The mean FPG for all participants was 4.7 mmol/L, and 1 and 2 h mean values were 7.0 and 6.0 mmol/L, respectively (Table 2 including Table 2 legend).

**Table 2:** Characteristics of maternal glucose metabolism by groups based on OGTT at 24–28 weeks’ gestation

	All	GDM_DK_	New-GDM	No-GDM
Participants% (*N*)	100.0% (465)	2.2% (10)	19.4% (90)	78.5% (365)
*Study OGTT 24–28 weeks’ gestation*				
*Venous plasma glucose (mmol/L)*				
Fasting	4.7 (0.4)	5.2 (0.5)	5.3 (0.3)	4.5 (0.3)
1-h	7.0 (1.7)	10.1 (1.0)	8.6 (1.6)	6.5 (1.3)
2-h	6.0 (1.3)	9.8 (0.8)	6.8 (1.1)	5.7 (1.1)
Gestational age at time of study OGTT (weeks)^a^	25.7 (24.7–27.1)	25.8 (24.6–29.3)	26.0 (24.7–27.5)	25.6 (24.7–27.0)
*Any glucose value above the diagnostic threshold*				
Fasting value ≥ 5.1 mmol/L^b^	18.5% (86)	50.0% (5)	90.0% (81)	–
1-h value ≥ 10.0 mmol/L	5.2% (24)	60.0% (6)	20.0% (18)	–
2-h value ≥ 8.5 mmol/L	3.9% (18)	100.0% (10)	8.9% (8)	–
2-h value ≥ 9.0 mmol/L	2.2% (10)	100.0% (10)	–	–
*Additional OGTTs in pregnancy*				
OGTT < 20 weeks	13.3% (62)	50.0% (5)	20.0% (18)	10.7% (39)
OGTT after study OGTT	10.3% (48)	–	23.3% (21)	7.4% % (27)
GDM_DK_ diagnosis at any time during pregnancy^c^	3.7% (17)	100.0% (10)	6.7% (6)	0.3% (1)
Insulin treatment for GDM_DK_	35.3% (6)	30.0% (3)	50.0% (3)	0% (0)

*OGTT* Oral glucose tolerance test, *GDM* Gestational diabetes mellitus, *GDM*_*DK*_ Women diagnosed with GDM by the Danish criterion (2 h glucose ≥ 9.0 mmol/L), *New-GDM* Women GDM_WHO2013_ positive and GDM_DK_ negative, *No-GDM* GDM_WHO2013_ and GDM_DK_ negative, *Additional OGTT* OGTT performed before or after the study OGTT as part of the Danish guidelines for GDM screening

Data are given as mean (SD) or percentages (N) unless otherwise stated

^a^Data are median (25–75 percentiles), as data were not normally distributed

^b^GDM_WHO2013_ diagnosis were based solely on a FPG ≥ 5.1 mmol/L for 14.4% (67/465) of all participants, 74.4% (67/90) of New-GDM-women and 0% (0) of GDM_DK_-women

^c^GDM_DK_ diagnosis by a 2 h value ≥ 9.0 mmol/L, and *N* = 1 based on pre- and postprandial glucose measurements and HbA1c due to failed completion of additional OGTT

The proportion of women who underwent an additional OGTT after the study OGTT was 23.3% (*N* = 21) among New-GDM-women compared to 7.4% (*N* = 27) among No-GDM-women (Table 2). From these additional OGTTs, 6.7% (*N* = 6) of the New-GDM-women and 0.3% (*N* = 1) of the No-GDM-women were diagnosed with GDM_DK_ (Table 2). The overall GDM_DK_ prevalence among study participants reached 3.7%, whereas the overall prevalence among the total number of women included for follow-up was 4.4%, which was driven by a slightly higher prevalence among non-participants (4.6%) (Table 2 and Online Resource 2).

### Maternal and neonatal outcomes

New-GDM-women had higher frequencies of pregnancy-induced hypertension, and their offspring more often had neonatal hypoglycaemia and were admitted to a NICU than offspring of the No-GDM-women. Despite being born significantly earlier than the infants of No-GDM mothers, New-GDM-infants had a higher birth weight z-score and were more often LGA. No difference was found regarding the remaining pregnancy outcomes, including induction of labour, caesarean delivery, birth weight and small-for-gestational-age infants (Table 3).

**Table 3:** Maternal and neonatal outcomes by groups based on OGTT at 24–28 weeks’ gestation

	New-GDM	No-GDM	*p* value ^a^
Number	90	365	
*Maternal outcomes*			
Pregnancy-induced hypertension	13.3% (12)	4.1% (15)	**.002**
Induction of labour	31.1% (28)	23.8% (87)	.177
Instrumental delivery	7.8% (7)	7.4% (27)	1.0
Caesarean delivery	28.9% (26)	18.6% (68)	.042
Postpartum haemorrhage (≥ 1,000 mL)	10.0% (9)	8.5% (31)	.679
3rd or 4th degree perineal/anal tears	5.6% (5)	2.7% (10)	.191
*Neonatal outcomes*^*b*^			
Gestational age at delivery (weeks)^c^	39.7 (38.7–40.7)	40.3 (39.3–41.0)	**.003**
Male infant sex	54.4% (49)	51.8% (189)	.814
Birth weight (g)	3,708 (523)	3,579 (513)	.037
Birth weight z-score^c^	0.43 (− 0.13–1.23)	− 0.11 (− 0.68–0.56)	** < .0001**
LGA infant (> 90^th^ percentile)	22.2% (20)	9.9% (36)	**.004**
SGA infant (< 10^th^ percentile)	2.2% (2)	7.7% (28)	.061
Abdominal circumference at birth (cm)	33.9 (1.9)	33.4 (2.0)	.040
Preterm delivery (< 37 weeks)	4.4% (4)	2.7% (10)	.492
Composite complication outcome	4.4% (4)	1.4% (5)	.084
Neonatal hypoglycaemia	8.9% (8)	1.9% (7)	**.004**
Neonatal intensive care unit Number of days admitted^c^	16.7% (15) 5.0 (2.0–8.0)	5.8% (21) 3.0 (2.0–6.0)	**.002** .770

*GDM* Gestational diabetes mellitus, *New-GDM* Women GDM_WHO2013_ positive and GDM_DK_ negative, *No-GDM* GDM_WHO2013_ and GDM_DK_ negative, *Birth weight z-score* Adjusted for gestational age and sex. *LGA* Large-for-gestational-age. *SGA* Small-for-gestational-age, *Composite complication outcome* Shoulder dystocia, nerve injury or bone fracture, *Neonatal hypoglycaemia* Defined as plasma glucose < 2.5 mmol/L at 2 h of life

Data are given as mean (SD) or percentages (N) unless otherwise stated

^a^*P* values are corrected for multiple testing using the Benjamini–Hochberg procedure, bold *p* values were considered significant as they were below the calculated critical value of .017

^b^For neonatal outcomes, data on multiple pregnancies are excluded (*N* = 3)

^c^Data are median (25–75 percentiles), as data were not normally distributed

**Fig. 2:**
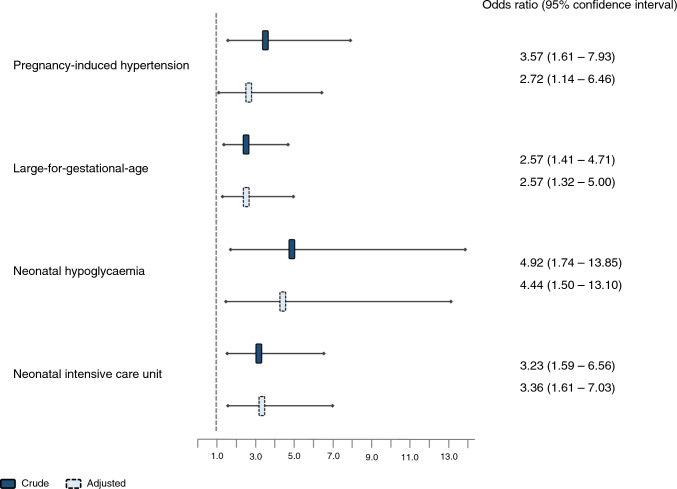
Odds ratios for adverse outcomes in New-GDM-women compared to No-GDM-women. Pregnancy-induced hypertension was adjusted for maternal BMI, age and chronic hypertension; large-for-gestational-age for maternal BMI, country of origin, educational level, smoking, parity and infant sex; neonatal hypoglycaemia for maternal BMI; and neonatal intensive care unit admission for maternal BMI and age

These findings were reflected in the exploratory crude logistic regression analyses, where the odds ratios (OR) for adverse maternal and neonatal outcomes were significantly increased among New-GDM-women, ORs ranging 2.57–4.92 but with wide confidence intervals (Fig. 2). The overall pattern was not changed in the adjusted analyses.

Maternal and neonatal outcomes were overall unchanged in a sensitivity analysis excluding the six New-GDM-women and the one No-GDM-woman who later in pregnancy were diagnosed with and treated for GDM_DK_ (Table 2 and Online Resource 3).

## Discussion

Using universal screening, WHO2013 diagnostic criteria resulted in a tenfold increase in GDM prevalence compared to the current Danish “2 h glucose only” criterion. This increase was mainly driven by higher FPG levels. Currently untreated women, whose glucose values lie in the gap between the higher Danish and the lower WHO2013 diagnostic criteria, had an increased risk of adverse pregnancy outcomes compared to women without GDM.

### Global changes in GDM prevalence and outcomes after implementation of WHO2013 criteria

Our finding of a higher GDM prevalence after the implementation of the WHO2013 criteria is in agreement with previous studies, even though globally, the changes in GDM prevalence have varied considerably, primarily due to differences in previous diagnostic criteria and screening practices [16, 17]. In European cohorts, the GDM prevalence is generally high when using WHO2013 criteria, ranging from 12.3 to 52.0% [5, 18, 19].

Despite some differences in OGTT methods and the previous diagnostic criteria used for comparison, previous studies have found higher rates of several adverse pregnancy outcomes in the untreated New-GDM-women compared to No-GDM-women [20–22]. Of these studies, only one identified significantly higher frequencies of neonatal hypoglycaemia and NICU admission for New-GDM-women compared to No-GDM-women as in the present study [20]. Partly, this may be explained by the New-GDM-women being defined from different previous diagnostic criteria and perhaps by population differences including ethnicity [23]. In contrast, other studies only identified a limited higher risk among the New-GDM-women [7, 24–26, 26]. A Danish study evaluating maternal and neonatal outcomes found little evidence of higher frequencies of adverse pregnancy outcomes among untreated New-GDM-women defined solely by the WHO2013 FPG threshold, which is opposite to the findings of significantly higher risk of adverse pregnancy outcomes in pregnancies of New-GDM-women of whom 74.4% had a FPG above threshold [7]. Although six women in the New-GDM-group and one in the No-GDM-group were diagnosed with GDM_DK_ after the study OGTT and thus received treatment, the sensitivity analysis comparing untreated women did not indicate that the effect of treatment skewed the results.

### Identification of new-GDM-women driven by the fasting value

As in the present study, other studies–though not all [27–30]–have also reported the increase in GDM prevalence to be driven by the relatively low WHO2013 FPG threshold [7, 31–37]. Two previous studies describing the FPG level in Danish pregnant women found high GDM prevalence driven by FPG values, supporting our findings. The OCC study identified 40% with an FPG ≥ 5.1 mmol/L and a tenfold increase in GDM prevalence based solely on the FPG threshold, and the other study including only obese pregnant women identified 39–44% with GDM based on the combination of FPG and 2 h WHO2013 thresholds [7, 37]. These findings indicate that Danish pregnant women constitute one of the populations with relatively high FPG levels. Accordingly, based on the OCC data, it has been proposed to use a higher FPG threshold of 5.6 mmol/L in Denmark.

Clearly, the FPG values vary across populations, however, such variations may not only be caused by population differences but also by pre-analytical differences, including sample handling and choice of sampling tubes [38]. In our study, blood samples were analysed prospectively using glucose-stabilising FC-Mix tubes. In contrast, in the HAPO study, the less glucose-stabilising fluoride-oxalate (FO-Mix) tubes were used, and analyses were performed on thawed biobank samples. If applying the suggested correction of − 0.2 mmol/L when using the more stabilised FC-Mix tubes compared to FO-Mix, the resulting mean FPG glucose in our study (4.7 mmol/L) would be identical to the HAPO cohort (4.5 mmol/L) [1, 39]. Still, the mean FPG in the present study was substantially lower than the OCC cohort (5.1 mmol/L), which was based on biobank analyses but using FC-Mix tubes—a difference for which we find no obvious explanation [7].

### Will it make a difference diagnosing women at a lower glycaemic level?

The current study cannot determine whether treating New-GDM-women diagnosed by WHO2013 would improve pregnancy outcomes. However, previous landmark RCTs have demonstrated effects of GDM treatment on several pregnancy complications, including LGA [40, 41]. In contrast, another “pragmatic” randomised trial of GDM screening from the USA found no difference in the population risk of complications [42].

In the recently published GEMS RCT from New Zealand, the effect of treatment was explored according to the higher New Zealand criteria and lower WHO2013 criteria [9]. On a population level, the study did not show a reduced risk of the primary outcome (LGA) with treatment between high and low criteria. However, treatment of the New-GDM-women resulted in significant reductions in LGA, shoulder dystocia and pre-eclampsia, with a more pronounced effect than previously reported and a number needed to treat as low as four to prevent one case of pre-eclampsia [40, 41]. These findings suggest that using WHO2013 criteria in a Danish population could potentially improve pregnancy outcomes for the currently untreated women with glucose values in the gap between the present Danish and the WHO2013 thresholds. The overall treatment effect in these women will hopefully be further clarified by the upcoming results from a Swedish RCT [43]. Nevertheless, the findings of more adverse pregnancy outcomes among New-GDM-women raise support for changing screening strategy and diagnostic criteria in Denmark.

### Strengths and limitations

This study is the first to evaluate the WHO2013 diagnostic criteria in a prospective Danish cohort. The sensitivity analysis, including data from the majority of eligible women, confirmed that the study participants were comparable to non-participants on most baseline characteristics, although non-participants had significantly fewer risk factors for GDM. Such difference may introduce selection bias towards increasing GDM prevalence and higher rates of adverse pregnancy outcomes among study participants, however, there were no differences between participants and non-participants regarding the overall GDM_DK_ prevalence or gestational age at diagnosis. The GDM_DK_ prevalence of 4.4% among all included for follow-up is lower than the overall prevalence in Denmark in 2020 of 5.9%. The cohort of women at Nordsjællands Hospital Hillerød therefore represent a subpopulation at lower risk [44, 45]. Thus, the external validity of the estimated GDM prevalence should be interpreted with caution, although a tenfold increase in GDM prevalence when implementing the fasting WHO2013 threshold has previously been reported in another Danish cohort [7].

Due to the COVID-19 pandemic, the final sample size was smaller than planned in the original power calculation. This power calculation was, however, conservative, and despite this cautious approach, we observed significant differences not only for the primary outcome but also for several secondary outcomes. However, in the logistic regression analyses, lack of power resulted in wide confidence intervals, which underlines the uncertainty of the risk estimates in addition to the uncertainty of the exploratory analyses in themselves.

## Conclusions

Introducing WHO2013 diagnostic criteria in a Danish cohort resulted in a tenfold increase in GDM prevalence, mainly driven by higher fasting glucose levels. Currently untreated women who lie in the gap between Danish and WHO2013 diagnostic criteria and their infants had higher risks of adverse pregnancy outcomes than women without GDM. The results indicate a potential for improving pregnancy outcomes and advocate for the necessity of changing diagnostic criteria in Denmark.

### Supplementary Information

Below is the link to the electronic supplementary material.

Supplementary file1 (DOCX 59 KB)

## Data Availability

The datasets generated and/or analysed during the current study are available from the corresponding author on reasonable request.
