# Supplementary material for: Applying WHO2013 diagnostic criteria for gestational diabetes mellitus reveals currently untreated women at increased risk
Source: Acta Diabetol. 2023 Jul 18;60(12):1663–73. doi: 10.1007/s00592-023-02148-2 (PMC10587026; doi:10.1007/s00592-023-02148-2)
Supplement: Supplementary file 1 — Supplementary file1 (DOCX 59 KB) [file 592_2023_2148_MOESM1_ESM.docx]

**Online Resource 1, 2 and 3 for the article “Applying WHO2013 diagnostic criteria for gestational diabetes reveals currently untreated women at increased risk”**

by Cathrine Munk Scheuer, Dorte Møller Jensen, H. David McIntyre, Lene Ringholm, Elisabeth Reinhardt Mathiesen, Celina Pforr Korsgård Nielsen, Rúna Louise Mortansdóttir Nolsöe, Julie Milbak, Thore Hillig, Peter Damm, Martin Overgaard and Tine Dalsgaard Clausen
in **Acta Diabetologica**

Corresponding author: Cathrine Scheuer cathrine.munk.scheuer.01@regionh.dk

**Online Resource 1: Benjamini-Hochberg procedure of correction for multiple testing**

The Benjamini-Hochberg (BH) procedure was used to correct for multiple testing in the two sets of secondary analyses comparing maternal and neonatal outcomes between groups, the comparison of New-GDM and No-GDM women (Table 3) and the sensitivity analysis of pregnancy outcomes among untreated women (Online Resource 3).

All original *p* values were ranked the lowest value given number 1, the second lowest value given number 2 etc. The BH equation *i/m*Q* was used to calculate the critical value for each original *p* value, *i* being the rank of *p* value, *m* being the total number of tests, and setting *Q* to 5% (table 1A and 1B).

The new corrected significance level was thus the critical value at which the largest original *p* value was smaller than its BH critical value, resulting in all original *p* values smaller than the new significance level being considered significant.

As displayed in tables 1A and 1B, the corrected significance levels were 0.017 for the comparisons of New-GDM and No-GDM women and 0.014 for the sensitivity analysis of pregnancy outcomes among untreated women.

| **Online Resource 1A: Benjamini-Hochberg procedure for New-GDM vs No-GDM (Table 3)** | | | |
| --- | --- | --- | --- |
| **Variable** | **Original *p* value** | **Rank (*i*)** | **BH critical value = *(i/m)*Q*** |
| Birth weight z-score ^b^ | **< .0001 *** | 1 | .003 |
| Pregnancy-induced hypertension | **.002 *** | 2 | .006 |
| Neonatal intensive care unit | **.002 *** | 3 | .008 |
| Gestational age at delivery (weeks) ^b^ | **.003 *** | 4 | .011 |
| Neonatal hypoglycaemia | **.004 *** | 5 | .014 |
| LGA infant (> 90^th^ percentile) | **.004 *** | 6 | .017 |
| Birth weight (g) | **.037** | 7 | .019 |
| Abdominal circumference at birth (cm) | **.040** | 8 | .022 |
| Caesarean delivery | **.042** | 9 | .025 |
| SGA infant (< 10^th^ percentile) | .061 | 10 | .028 |
| Composite complication outcome | .084 | 11 | .031 |
| Induction of labour | .177 | 12 | .033 |
| 3^rd^ or 4^th^ degree perineal/anal tears | .191 | 13 | .036 |
| Preterm delivery (< 37 weeks) | .492 | 14 | .039 |
| Postpartum haemorrhage (≥1,000 mL) | .679 | 15 | .042 |
| Number of days admitted ^c^ | .770 | 16 | .044 |
| Male infant sex | .814 | 17 | .047 |
| Instrumental delivery | 1.0 | 18 | .050 |
| Bold p values were significant in the original version.  * P values still significant after Benjamini-Hochberg correction for multiple testing.  The BH critical value is 0.017, as 0.004 is the highest original *p* value that is smaller than the corresponding BH critical value. | | | |

| **Online Resource 1B: Benjamini-Hochberg procedure for Untreated New-GDM vs No-GDM (Online Resource 2)** | | | |
| --- | --- | --- | --- |
| **Variable** | **Original *p* value** | **Rank (*i*)** | **BH critical value = *(i/m)*Q*** |
| Birth weight z-score | **< .0001 *** | 1 | .003 |
| Pregnancy-induced hypertension | **.001 *** | 2 | .006 |
| Neonatal intensive care unit | **.003 *** | 3 | .008 |
| LGA infant (> 90^th^ percentile) | **.008*** | 4 | .011 |
| Gestational age at delivery (weeks) | **.013 *** | 5 | .014 |
| Neonatal hypoglycaemia | **.022** | 6 | .017 |
| Birth weight (g) | **.023** | 7 | .019 |
| SGA infant (< 10^th^ percentile) | **.026** | 8 | .022 |
| Abdominal circumference at birth (cm) | **.049** | 9 | .025 |
| Caesarean delivery | .052 | 10 | .028 |
| Composite complication outcome | .071 | 11 | .031 |
| 3^rd^ or 4^th^ degree perineal/anal tears | .174 | 12 | .033 |
| Induction of labour | .264 | 13 | .036 |
| Preterm delivery (< 37 weeks) | .313 | 14 | .039 |
| Male infant sex | .628 | 15 | .042 |
| Instrumental delivery | .820 | 16 | .044 |
| Postpartum haemorrhage (≥1,000 mL) | .830 | 17 | .047 |
| Number of days admitted | .881 | 18 | .050 |
| Bold p values were significant in the original version.  * P values still significant after Benjamini-Hochberg correction for multiple testing.  The BH critical value is 0.014, as 0.013 is the highest original *p* value that is smaller than the corresponding BH critical value. | | | |

| **Online Resource 2: Baseline characteristics of study participants compared to non-participants** | | | | |
| --- | --- | --- | --- | --- |
|  | **Included for follow-up** | **Study participants** | **Non-participants ^a^** | ***p* value ^b^** |
| Number | 1,626 | 465 | 1,161 |  |
| **Baseline characteristics** |  |  |  |  |
| Age at delivery (years) | 32.1 (4.8) | 31.9 (4.5) | 32.1 (5.0) | .404 |
| Risk factors for GDM |  |  |  |  |
| GDM in previous pregnancy  Body mass index ≥27 kg/m^2^  Previous birth of child ≥4,500 g  Family history of diabetes  Polycystic ovarian syndrome  Multiple pregnancy  Glucosuria | 1.4 % (22)  25.1 % (406)  1.7 % (27)  32.8 % (534)  3.0 % (49)  0.8 % (13)  5.1 % (83) | 0.9 % (4)  26.6 % (123)  2.2 % (10)  39.8 % (185)  3.7 % (17)  0.9 % (4)  8.0 % (37) | 1.6 % (18)  24.5 % (283)  1.5 % (17)  30.1 % (349)  2.8 % (32)  0.8 % (9)  4.0 % (46) | .348  .410  .390  **< .0001**  .338  1.0  **.002** |
| ≥ 1 risk factor for GDM | 57.8 % (937) | 63.7 % (296) | 55.4 % (641) | **.003** |
| ≥ 2 risk factors for GDM | 19.3 % (313) | 19.6 % (91) | 19.2 % (222) | .889 |
| Pre-gestational body mass index (kg/m^2^) ^c^ | 23.4 (21.1 – 27.0) | 23.7 (21.3 – 27.1) | 23.3 (21.0 – 26.8) | **.043** |
| Parous (≥ 1 prior births) | 64.9 % (1,055) | 61.3 % (285) | 66.4 % (770) | .058 |
| Country of origin: Denmark | 78.5 % (1,190) | 81.2 % (366) | 77.4 % (824) | .116 |
| Married / de-facto relationship | 96.6 % (1,556) | 97.6 % (451) | 96.2 % (1,105) | .173 |
| Current smoker | 4.6 % (75) | 3.7 % (17) | 5.0 % (58) | .295 |
| Chronic hypertension | 0.7% (11) | 0.6 % (3) | 0.7 % (8) | 1.0 |
| **GDM screening and diagnosis** |  |  |  |  |
| Number of OGTTs  < 20 weeks  > 20 weeks ^d^  Total number of OGTTs in pregnancy | 10.9 % (177)  59.7 % (971)  0.8 (0.8) | 13.3 % (62)  100.0 % (465)  1.3 (0.6) | 9.9 % (115)  43.6 % (506)  0.6 (0.7) | .052  **< .0001**  **< .0001** |
| GDM_DK_ diagnosis ^e^  < 20 weeks  > 20 weeks  Any time during pregnancy | 0.7 % (12)  3.6 % (59)  4.4 % (71) | –  3.7 % (17)  3.7 % (17) | 1.0 % (12)  3.6 % (42)  4.6 % (54) | N/A  1.0  .422 |
| Gestational age at time of GDM_DK_ diagnosis (weeks) ^c^ | 28.0 (24.0 – 33.0) | 29.0 (25.5 – 34.5) | 28.0 (21.9 – 32.0) | .193 |
| GDM: Gestational Diabetes Mellitus. OGTT: Oral Glucose Tolerance Test. N/A: Not applicable.  Data are given as mean (SD) or percentages (N) unless otherwise stated.  ^a^ Non-participants defined as women who were included for follow-up but did not complete the study OGTT (N=754+872–465).  ^b^ *p* values compare Study participants and Non-participants.  ^c^ Data are median (25-75 percentiles), as data were not normally distributed.  ^d^ Including study OGTTs.  ^e^ GDM_DK_ diagnosis by a 2-hour value ≥ 9.0 mmol/L, and N=3 few based on pre- and postprandial glucose measurements and HbA1c due to failed completion of additional OGTT.  Missing data for the whole cohort (N=1,626) summed up to: Date of delivery N=20; Due date N=1; Pre-pregnancy body mass index N=9; Family history of diabetes N=111; Polycystic ovarian syndrome N=13; Married/de-facto relationship N=14; Parity N=1; Country of origin N=110. | | | | |

| **Online Resource 3: Maternal and neonatal outcomes of untreated women ^a^** | | | |
| --- | --- | --- | --- |
|  | **Untreated New-GDM** | **Untreated No-GDM** | ***p* value ^b^** |
| Number | 84 | 364 |  |
| **Maternal outcomes** |  |  |  |
| Pregnancy-induced hypertension | 14.3 % (12) | 3.9 % (14) | **.001** |
| Induction of labour | 29.8 % (25) | 23.8 % (86) | .264 |
| Instrumental delivery | 8.3 % (7) | 7.5 % (27) | .820 |
| Caesarean delivery | 28.6 % (24) | 18.8 % (68) | .052 |
| Postpartum haemorrhage (≥ 1,000 mL) | 9.5 % (8) | 8.6 % (31) | .830 |
| 3^rd^ or 4^th^ degree perineal/anal tears | 6.0 % (5) | 2.8 % (10) | .174 |
| **Neonatal outcomes ^c^** |  |  |  |
| Gestational age at delivery (weeks) ^d^ | 39.8 (38.9 – 40.7) | 40.3 (39.3 – 41.0) | **.013** |
| Male infant sex | 56.0 % (47) | 52.6 % (189) | .628 |
| Birth weight (g) | 3,725 (517) | 3,581 (511) | .023 |
| Birth weight z-score ^d^ | 0.42 (-0.12 – 1.21) | -0.11 (-0.68 – 0.56) | **< .0001** |
| LGA infant (> 90^th^ percentile) | 21.4 % (18) | 10.0 % (36) | **.008** |
| SGA infant (< 10^th^ percentile) | 1.2 % (1) | 7.5 % (27) | .026 |
| Abdominal circumference at birth (cm) | 33.9 (1.9) | 33.4 (2.0) | .049 |
| Preterm delivery (< 37 gestational weeks) | 4.8 % (4) | 2.8 % (10) | .313 |
| Composite complication outcome | 4.8 % (4) | 1.4 % (5) | .071 |
| Neonatal hypoglycaemia | 7.1 % (6) | 1.9 % (7) | .022 |
| Neonatal intensive care unit  Number of days admitted ^d^ | 16.7 % (14)  5.0 (1.8 – 8.0) | 5.8 % (21)  3.0 (2.0 – 6.0) | **.003**  .881 |
| GDM: Gestational Diabetes Mellitus. New-GDM: Women GDM_WHO2013_ positive and GDM_DK_ negative. No-GDM: GDM_WHO2013_ and GDM_DK_ negative. Birth weight z-score: Adjusted for gestational age and sex. LGA: Large-for-gestational-age. SGA: Small-for-gestational-age. Composite complication outcome: Shoulder dystocia, nerve injury or bone fracture. Neonatal hypoglycaemia: Defined as plasma glucose <2.5 mmol/L at 2 hours of life.  Data are given as mean (SD) or percentages (N) unless otherwise stated.  ^a^ After the study OGTT, 6 New-GDM-women and 1 No-GDM-women were diagnosed with GDM by Danish criteria and therefore treated in accordance with clinical guidelines. This sensitivity analysis includes only those in the two groups that remained untreated during pregnancy.  ^b^ *P* values are corrected for multiple testing using the Benjamini-Hochberg procedure, bold *p* values were considered significant as they were below the calculated critical value of .014.  ^c^ For neonatal outcomes, data on multiple pregnancies are excluded (N=3).  ^d^ Data are median (25-75 percentiles), as data were not normally distributed. | | | |
